# Gender-Related Difference in D-Dimer Level Predicts In-Hospital Heart Failure after Primary PCI for ST-Segment Elevation Myocardial Infarction

**DOI:** 10.1155/2021/7641138

**Published:** 2021-08-10

**Authors:** Li Li, Wei Wang, Tai Li, Ying Sun, Yanjun Gao, Lin Wang, Heng-Chen Yao

**Affiliations:** ^1^Department of Cardiology, Liaocheng People's Hospital, Liaocheng 252000, China; ^2^Department of Nursing, Liaocheng Vocational & Technical College, Liaocheng 252000, China; ^3^Department of Clinical Laboratory, Liaocheng People's Hospital, Liaocheng 252000, China; ^4^Cardiologic Color Doppler Room, Liaocheng People's Hospital, Liaocheng 252000, China

## Abstract

**Aims:**

The prognostic value of plasma D-dimer in patients with coronary artery disease (CAD) remains controversial. The study is aimed at investigating the relationship between plasma D-dimer levels and in-hospital heart failure (HF) in ST-segment elevation myocardial infarction (STEMI) patients who underwent primary percutaneous coronary intervention (pPCI).

**Methods:**

STEMI patients who underwent pPCI were enrolled in this study. Venous blood samples were collected from patients on admission before pPCI procedure. The study endpoint was the occurrence of in-hospital HF. The participants were divided into two groups according to plasma D-dimer levels and further compared baseline D-dimer levels between male and female. Logistic regression and receiver operating characteristic (ROC) curves were performed to evaluate the relationship of D-dimer and in-hospital HF.

**Results:**

A total of 778 patients were recruited in the study, of which 539 (69.3%) patients had normal D-dimer levels (≤0.5 mg/L) while 239 (30.7%) had increased D-dimer levels (>0.5 mg/L). The female patients have higher D-dimer levels and higher incident rate of in-hospital HF than that in male patients (*p* < 0.001). The multivariate logistic regression model revealed that D-dimer was an independent predictor for in-hospital HF in overall population (adjusted odds ratio [OR]: 1.197, 95% CI: 1.003-1.429, and *p* = 0.046) and female patients (adjusted OR: 1.429, 95% CI: 1.083-1.885, and *p* = 0.012).

**Conclusion:**

Increased plasma D-dimer levels were an independent risk factor for incidence of in-hospital HF in STEMI patients who underwent pPCI, especially in female patients, which provides guidance for clinicians in identifying patients at high risk of developing HF and lowering their risk.

## 1. Introduction

D-dimer is a biomarker of thrombosis and hypercoagulability; it can be measured in plasma or whole blood [1, 2]. Elevated plasma levels of D-dimer have been proven to be associated with subsequent thrombotic events and are extensively used to diagnose venous thromboembolism (VTE) [3–5]. Previous studies showed that D-dimer levels could predict adverse outcomes, including fatal events in patients with coronary heart disease (CHD), in terms of both short-term outcomes and long-term follow-up [5–10].

Heart failure (HF) following myocardial infarction (MI) is common and may develop during hospitalization; it may even be present on admission [11]. Furthermore, HF is the strongest predictor of death and has a great implication for treating patients with acute MI, especially women [12]. Previous studies demonstrated that D-dimer levels were more predictive for cardiac events in patients with acute MI; however, HF was not included [8, 13, 14]. To date, the role of D-dimer level in predicting in-hospital HF in patients with ST-segment elevation MI (STEMI) is not clear, particularly after primary percutaneous coronary intervention (pPCI). Moreover, few reports investigated the gender difference in the relationship between D-dimer levels and the incidence of HF in patients with acute MI.

Thus, the present analysis was performed to evaluate the possible relationship between D-dimer levels and incidence of in-hospital HF in patients with STEMI undergoing pPCI in the overall population and in subgroups with gender difference.

## 2. Methods

### 2.1. Study Design and Patient Selection

**Figure 1:**
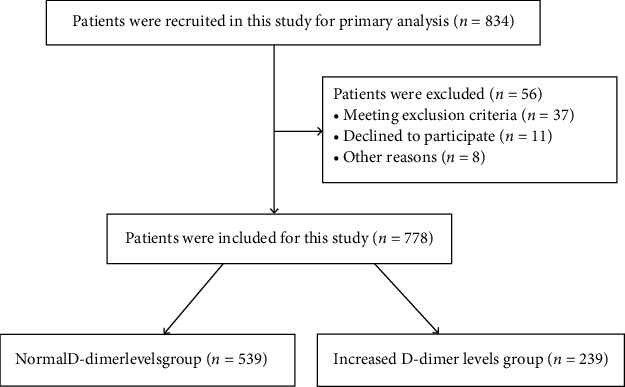
The flowchart of studied patients.

Consecutive patients with STEMI who underwent pPCI at Liaocheng People's Hospital from June 2015 to November 2019 were enrolled in this study. Acute STEMI was defined as follows: chest pain typically lasts more than 30 minutes, and ST-segment elevation of adjacent two electrocardiography leads at least 2 mm within 12 hours of symptom onset [15]. The symptom onset could be prolonged to 24 hours if evidence of hemodynamic instability or persistent ischemia was existing [10]. Patients diagnosed with STEMI who underwent pPCI with stent implantation were included in the study. The exclusion criteria were as follows: severe infections, severe liver and renal diseases, aortic dissection, immune system diseases, cancer, and severe complications during the procedure. Patients with HF at admission were also excluded. A total of 778 patients were included in this study. The study flowchart is shown in Figure 1. All the protocols were approved by the Liaocheng People's Hospital's Ethics Committee, and informed consent forms were obtained from all patients.

### 2.2. Primary PCI

PPCI procedures were performed by experienced cardiologists. All patients received standard loaded dose medications (300 mg of aspirin and 300 mg clopidogrel or 180 mg ticagrelor) upon the diagnosis of STEMI. After successful puncture, all patients were administrated 2500 IU heparin, with a weight dependent dose (up to 100 IU/kg) added for PCI. All decisions and procedures, including radial/femoral approach and intervention strategy, were determined by the operator's discretion.

### 2.3. Laboratory Testing and Echocardiography

All venous blood samples were collected from patients on admission before the pPCI procedure. All the laboratory tests were performed by the emergency laboratory of our hospital. The plasma levels of D-dimer were measured by the immunoturbidimetry method using HemosIL D-dimer HS 500 kit (Werfen, Barcelona, Spain) according to the manufacturer's instruction, and ≤0.5 mg/L was defined as normal. Echocardiography was performed within 24 hours on admission to determine left ventricular ejection fraction (LVEF).

### 2.4. Study Endpoints

Patients were evaluated by an attending cardiologist. According to clinical observations, the definition of HF was mainly based on ESC guidelines for the diagnosis and treatment of acute and chronic HF [16]. HF was defined as the presence of symptoms of breathlessness and clinical signs of pulmonary congestion on physical examination, including an S3 gallop, pulmonary or alveolar rales, and/or interstitial edema during a hospital stay.

### 2.5. Statistical Analysis

Statistical analysis was performed using the SPSS software, version 23.0 (IBM Corp.). Continuous data with normal distribution were expressed as mean ± standard deviation and compared using the Student *t*-test. Data with nonnormal distribution were expressed as median (interquartile range) and compared using the Mann­Whitney *U* test. Categorical variables were expressed as frequency and percentage and compared using the Chi-square test or Fisher exact test. Logistic regression analysis was used to determine whether D-dimer was an independent predictor of in-hospital HF in the total study population, male and female, respectively. Variables such as age, female gender, previous history of smoking, hypertension, and DM were identified with univariate analysis, and the unadjusted *p* value <0.05 in the univariate analysis was subsequently evaluated using a multivariate logistic regression model. Receiver operating characteristic (ROC) curves were used to determine the best cut-off values of D-dimer in predicting in-hospital HF in the total study population, male and female, respectively. A *p* value <0.05 was considered to be statistically significant.

## 3. Results

### 3.1. Patient Characteristics

From June 2015 to November 2019, 834 patients underwent pPCI and 778 patients were included in the study finally. Of the 778 patients, 539 (69.3%) had normal D-dimer levels (≤0.5 mg/L) and 239 (30.7%) had increased D-dimer levels (>0.5 mg/L). The baseline characteristics of the two groups are shown in Table 1. Patients with increased D-dimer levels were older (*p* < 0.001) and nonsmokers (*p* = 0.032) and had a higher incidence of in-hospital HF (*p* < 0.001).

In the subgroup analysis, all patients were divided into two groups according to gender: male (582, 74.8%) group and female (196, 25.2%) group (as shown in Table 2). The plasma levels of D-dimer (*p* < 0.001) and the incidence of in-hospital HF (*p* < 0.001) were higher in female patients than in male patients.

### 3.2. Multivariate Logistic Regression Model for In-Hospital HF

For in-hospital HF in the overall population, the result of the multivariate logistic regression revealed that D-dimer was an independent predictor of in-hospital HF in the overall population [adjusted odds ratio (OR): 1.197, 95% confidence interval (CI): 1.003-1.429, and *p* = 0.046]. The results showed that variables such as age, heart rate, and white blood cell (WBC) count were also independent factors of in-hospital HF (Table 3).

For in-hospital HF in the male population, the multivariate logistic regression model variables included heart rate, WBC count, and creatinine. The result revealed that the heart rate, but not D-dimer, was an independent predictor of in-hospital HF (Table 4). In the female population, the multivariate logistic regression model included WBC count, total cholesterol (TC), and D-dimer. The result revealed that D-dimer was an independent predictor of in-hospital HF [adjusted OR: 1.429, 95% CI: 1.083­-1.885, and *p* = 0.012] (Table 5).

### 3.3. ROC Curves for In-Hospital HF

**Figure 2:**
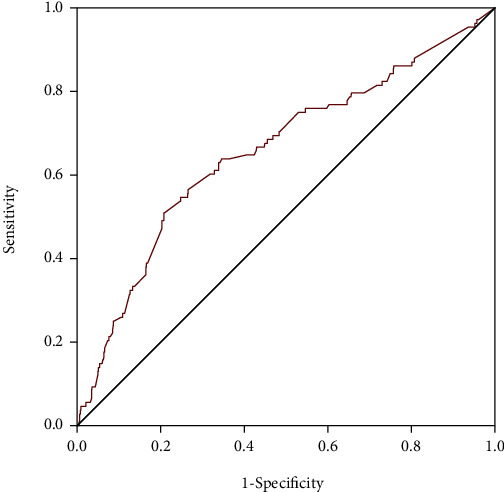
Receiver operating characteristic curves of D-dimer for predicting in-hospital heart failure in all patients.

**Figure 3:**
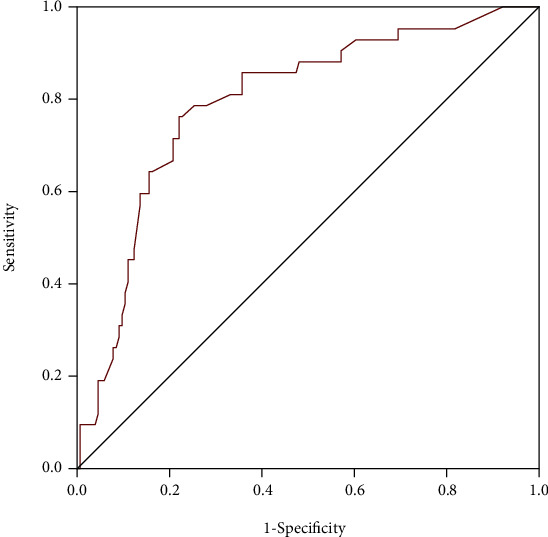
Receiver operating characteristic curves of D-dimer for predicting in-hospital heart failure in female patients.

**Figure 4:**
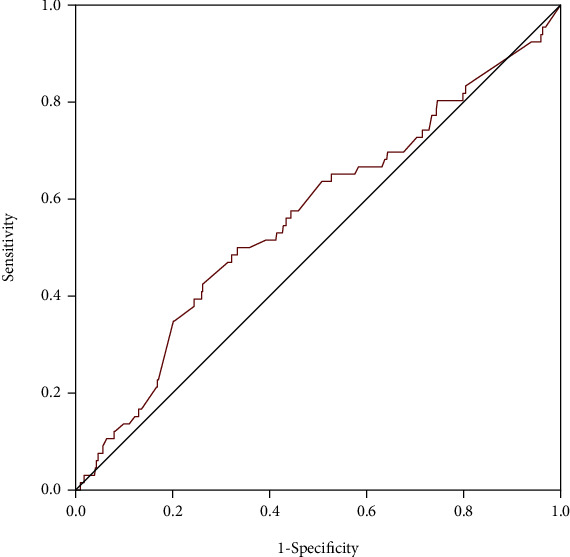
Receiver operating characteristic curves of D-dimer for predicting in-hospital heart failure in male patients.

ROC curves were performed to evaluate the potential predictive power of D-dimer for in-hospital HF in all patients, female and male, respectively (Figures 2–4). The area under the curve (AUC) values for D-dimer in predicting HF were 0.793 with 76% sensitivity and 78% specificity (*p* < 0.001) in female patients, 0.657 with 50.9% sensitivity and 79.3% specificity (*p* < 0.001) in the total population, and 0.565 with 50% sensitivity and 67% specificity (*p* = 0.085) in male patients. The results of the ROC curve analysis indicated that D-dimer had moderate predictive efficiency for predicting in-hospital HF in female patients and power superior to that in the total population and male patients. The performances of D-dimer in predicting in-hospital HF were shown in Table 6.

## 4. Discussion

This prospective study found that the D-dimer level was an independent predictor of in-hospital HF in patients with STEMI who underwent pPCI. In the subgroup analysis, D-dimer was an independent risk factor of in-hospital HF in female patients but not in male patients.

Several studies evaluated the value of D-dimer in predicting adverse outcomes in patients with coronary artery disease (CAD). The most robust evidence from the LIPID study demonstrated that D-dimer levels could independently predict long-term major cardiovascular mortality in patients with stable CAD [8]. The data from the AtheroGene study unearthed that the D-dimer level was an independent predictor of subsequent cardiovascular death in patients with CAD [14]. Moss et al. [17] uncovered that the elevated levels of D-dimer contributed independently to recurrent coronary events in postinfarction patients. Moreover, accumulating evidence has shown that the elevated levels of D-dimer can predict adverse outcomes including death in patients with acute coronary syndrome (ACS) [6, 10, 18, 19]. Patients with acute ischemic cerebrovascular disease complicating CHD, whose D-dimer levels were higher than the normal standard, had the worst outcomes [20]. These results were equally indicated that higher levels of D-dimer were associated with a higher incidence of major adverse cardiovascular events (MACEs). However, a retrospective study on 569 patients with STEMI, who underwent pPCI and were prospectively followed up for a median of 38 months, showed that D-dimer has no independent prognostic value in patients with STEMI [21]. Thus, the value of D-dimer in predicting adverse outcomes for CAD was still controversial. Furthermore, the association between D-dimer levels and the incidence of HF was not explored in the aforementioned studies. Recently, D-dimer was found to be associated with the incidence of HF after hospitalization in patients with AMI [22]. Furthermore, in the subsequent LIPID HF risk-prediction model, the results revealed that a high level of D-dimer was an independent predictor of incident HF in patients with ACS [23]. More importantly, one multicenter study showed that D-dimer levels were associated with the incidence of HF in adults from the general population [24]. Briefly, the present study results were in accordance with the findings of previous studies, and our study first time revealed the association in the pPCI population and further elucidated the gender-related difference in D-dimer predicting HF. Further analysis with longer follow-up might help clarify whether high D-dimer levels are an independent predictor of MACEs, especially HF.

A previous study showed that increased levels of D-dimer were significantly associated with myocardial necrosis and inversely correlated with LVEF after AMI [25]. Also, high D-dimer levels on admission were associated with a larger myocardial infarct size assessed using magnetic resonance imaging (MRI) data in patients treated with pPCI for STEMI [26]. Furthermore, multiple pathways such as inflammation [27], neurohormonal activation [28], and stagnation and endothelial dysfunction [29] were associated with the occurrence of HF, which also resulted in D-dimer elevation. More important, patients with HF were at risk of coagulation disorders and vascular thromboembolic events [30]. These mechanisms might explain the value of D-dimer in independently predicting the incidence of HF in STEMI patients undergoing pPCI.

## 5. Limitations

This study had several limitations. It was a single-center study. Although the sample size was relatively large, the study observation time was limited to the length of in-hospital stay; therefore, a long-term prognostic follow-up is warranted. Finally, data such as culprit lesion characteristics, total ischemic time, and procedural details were not collected and analyzed in this study.

## 6. Conclusions

In this prospective cohort study, increased levels of D-dimer were an independent risk factor of in-hospital HF in patients with STEMI who underwent pPCI, especially women. This analysis highlighted the importance of D-dimer and provided guidance for clinicians in identifying patients at high risk of developing HF and lowering their risk.

## Figures and Tables

**Table 1:** Basic characteristics of overall population by D-dimer level.

Variable	D − dimer ≤ 0.5 mg/L	D − dimer > 0.5 mg/L	*p* value
	*n* = 539	*n* = 239	
Age (years)	61 (17)	65 (16)	<0.001
Smoking, *n* (%)	302 (56.0)	114 (47.7)	0.032
Hypertension, *n* (%)	290 (53.8)	128 (53.5)	0.949
DM, *n* (%)	141 (26.2)	63 (26.4)	0.953
Heart rate (bpm)	76 (20)	78 (20)	0.233
Hemoglobin (g/dL)	144 (20)	139 (24)	<0.001
WBC count (×109/L)	9.67 (3.83)	9.97 (4.30)	0.281
PLT (×109/L)	231 (74)	221 (85)	0.064
Cr (*μ*mol/L)	62 (21)	64 (19)	0.002
TC (mmol/L)	4.78 (1.32)	4.64 ± 0.99	0.033
TG (mmol/L)	1.52 (1.26)	1.35 (1.00)	0.006
EF (%)	50 (9)	49 (11)	0.005
D-dimer (mg/L)	0.23 (0.23)	0.88 (0.60)	<0.001
Clopidogrel, *n* (%)	114 (21.2)	50 (20.9)	0.942
Ticagrelor, *n* (%)	425 (78.8)	189 (79.1)	0.942
In-hospital HF, *n* (%)	47 (8.7)	61 (25.5)	<0.001

DM: diabetes mellitus; WBC: white blood cell; PLT: platelet; Cr: creatinine; TC: total cholesterol; TG: triglyceride; EF: ejection fraction; HF: heart failure.

**Table 2:** Basic characteristics of patients with gender difference.

Variable	All patients	Man	Woman	*p* value
	*n* = 778	*n* = 582	*n* = 196	
Age (years)	62 (17)	59 (17)	67 (10)	<0.001
Smoking, *n* (%)	416 (53.5)	405 (69.6)	11 (5.6)	<0.001
Hypertension, *n* (%)	418 (53.7)	305 (52.4)	113 (57.6)	0.203
DM, *n* (%)	204 (26.2)	138 (23.7)	66 (33.7)	0.006
Heart rate (bpm)	76 (21)	76 (21)	76 (20)	0.552
Hemoglobin (g/dL)	143 (21)	147 (18)	131 (19)	<0.001
WBC count (×109/L)	9.8 (3.97)	9.95 (4.14)	9.55 (3.71)	0.083
PLT (×109/L)	228 (77)	224 (77)	240 (85)	0.025
Cr (*μ*mol/L)	62 (20)	66 (19)	53 (17)	<0.001
TC (mmol/L)	4.70 (1.31)	4.64 (1.21)	5.10 ± 1.51	<0.001
TG (mmol/L)	1.47 (1.23)	1.47 (1.26)	1.44 (1.12)	0.706
EF (%)	50 (10)	50 (9)	50 ± 10	0.368
D-dimer (mg/L)	0.31 (0.43)	0.30 (0.43)	0.40 (0.58)	<0.001
Clopidogrel, *n* (%)	164 (21.1)	115 (19.7)	49 (25.0)	0.120
Ticagrelor, *n* (%)	614 (78.9)	467 (80.3)	147 (75.0)	0.120
In-hospital HF, *n* (%)	108 (13.8)	66 (11.3)	42 (21.4)	<0.001

DM: diabetes mellitus; WBC: white blood cell; PLT: platelet; Cr: creatinine; TC: total cholesterol; TG: triglyceride; EF: ejection fraction; HF: heart failure.

**Table 3:** Logistic regression analysis of predictors of in-hospital heart failure in the overall population.

Variables	OR	95% CI	*p* value	Adjusted OR	95% CI	*p* value
Age	1.033	1.014-1.053	0.001	1.036	1.014-1.059	0.001
Gender	2.132	1.392-3.267	0.001	1.101	0.612-1.980	0.748
Smoking	1.816	1.201-2.744	0.005	1.246	0.736-2.112	0.413
Hypertension	1.627	1.068-2.479	0.023	1.569	1.006-2.448	0.047
Diabetes mellitus	1.648	1.070-2.539	0.023	1.484	0.936-2.354	0.093
Heart rate	1.019	1.006-1.031	0.003	1.022	1.009-1.035	0.001
Hemoglobin	0.982	0.971-0.994	0.002	0.985	0.971-1.000	0.052
WBC count	1.107	1.043-1.175	0.001	1.131	1.058-1.210	<0.001
PLT	1.000	0.997-1.003	0.863			
Cr	1.010	0.999-1.021	0.074			
TC	0.880	0.721-1.073	0.205			
TG	0.903	0.764-1.068	0.234			
D-dimer	1.359	1.159-1.594	<0.001	1.197	1.003-1.429	0.046

OR: odds ratio; CI: confidence interval; WBC: white blood cell; PLT: platelet; Cr: creatinine; TC: total cholesterol; TG: triglyceride; D-D: D-dimer.

**Table 4:** Logistic regression analysis of predictors of in-hospital heart failure in the male population.

Variables	OR	95% CI	*p* value	Adjusted OR	95% CI	*p* value
Age	1.021	0.999-1.045	0.065			
Smoking	1.257	0.732-2.158	0.406			
Hypertension	1.565	0.925-2.650	0.095			
Diabetes mellitus	1.466	0.835-2.577	0.183			
Heart rate	1.025	1.009-1.041	0.002	1.022	1.006-1.038	0.007
Hemoglobin	0.989	0.974-1.006	0.200			
WBC count	1.098	1.022-1.179	0.011	1.072	0.995-1.155	0.066
PLT	1.001	0.997-1.004	0.795			
Cr	1.018	1.005-1.032	0.005	1.018	1.004-1.031	0.010
TC	0.950	0.741-1.218	0.687			
TG	0.929	0.763-1.130	0.459			
D-dimer	1.122	0.861-1.463	0.395			

OR: odds ratio; CI: confidence interval; WBC: white blood cell; PLT: platelet; Cr: creatinine; TC: total cholesterol; TG: triglyceride; D-D: D-dimer.

**Table 5:** Logistic regression analysis of predictors of in-hospital heart failure in the female population.

Variables	OR	95% CI	*p* value	Adjusted OR	95% CI	*p* value
Age	1.042	0.998-1.087	0.061			
Smoking	2.847	0.354-22.898	0.325			
Hypertension	1.624	0.794-3.322	0.185			
Diabetes mellitus	1.656	0.823-3.335	0.158			
Heart rate	1.009	0.989-1.031	0.379			
Hemoglobin	0.987	0.965-1.009	0.251			
WBC count	1.180	1.049-1.327	0.006	1.143	1.009-1.295	0.035
PLT	0.999	0.994-1.004	0.652			
Cr	1.018	0.995-1.042	0.128			
TC	0.635	0.446-0.904	0.012	0.671	0.461-0.977	0.037
TG	0.846	0.605-1.184	0.330			
D-dimer	1.575	1.189-2.086	0.002	1.429	1.083-1.885	0.012

OR: odds ratio; CI: confidence interval; WBC: white blood cell; PLT: platelets; Cr: creatinine; TC: total cholesterol; TG: triglyceride; D-D: D-dimer.

**Table 6:** Performance of gender for D-dimer in prediction in-hospital heart failure.

	AUC	95% CI	*p* value	Sensitivity	Specificity	Cut-off
Total	0.657	0.597-0.716	<0.001	50.9%	79.3%	0.615
Male	0.565	0.488-0.643	0.085	50%	67%	0.443
Female	0.793	0.717-0.869	<0.001	76%	78%	0.615

AUC: area under the curve; CI: confidence interval population.

## Data Availability

The data used to support the findings of this study are available from the corresponding author upon request.
